# Detection of Oral Dysplastic and Early Cancerous Lesions by Polarization-Sensitive Optical Coherence Tomography

**DOI:** 10.3390/cancers12092376

**Published:** 2020-08-22

**Authors:** Ping-Hsien Chen, Hung-Yi Lee, Yi-Fen Chen, Yi-Chen Yeh, Kuo-Wei Chang, Ming-Chih Hou, Wen-Chuan Kuo

**Affiliations:** 1 Department of Gastroenterology, West Garden Hospital, Taipei 108, Taiwan; 2294@westgarden.com.tw; 2 Endoscopy Center for Diagnosis and Treatment, Taipei Veterans General Hospital, Taipei 112, Taiwan; 3 Department of Medicine, National Yang-Ming University, Taipei 112, Taiwan; ycyeh2@vghtpe.gov.tw (Y.-C.Y.); mchou@vghtpe.gov.tw (M.-C.H.); 4 Institute of Biophotonics, National Yang-Ming University, Taipei 112, Taiwan; a0987136909@ym.edu.tw; 5 Institute of Oral Biology, National Yang-Ming University, Taipei 112, Taiwan; u39917002@ym.edu.tw (Y.-F.C.); ckcw@ym.edu.tw (K.-W.C.); 6 Department of Pathology and Laboratory Medicine, Taipei Veterans General Hospital, Taipei 112, Taiwan; 7 Department of Dentistry, National Yang-Ming University, Taipei 112, Taiwan; 8 Department of Stomatology, Taipei Veterans General Hospital, Taipei 112, Taiwan; 9 Department of Internal Medicine, Taipei Veterans General Hospital, Taipei 112, Taiwan

**Keywords:** oral cancer, dysplasia, diagnosis, polarization-sensitive optical coherence tomography

## Abstract

Detection of oral dysplastic and early-stage cancerous lesions is difficult with the current tools. Half of oral cancers are diagnosed in a late stage. Detection of early stromal change to predict malignant transformation is a new direction in the diagnosis of early-stage oral cancer. The application of new optical tools to image stroma in vivo is under investigation, and polarization-sensitive optical coherence tomography (PS-OCT) is potentially one of those tools. This is a preliminary study to sequentially image oral stromal changes from normal, hyperplasia, and dysplasia to early-stage cancer by PS-OCT in vivo. We used 4-Nitroquinoline-1-oxide drinking water to induce dysplasia and early-stage oral cancer in 19 K14-EGFP-miR-211-GFP transgenic mice. A total of 8 normal, 12 hyperplastic, 11 dysplastic, and 4 early-stage cancerous lesions were enrolled. A new analytic process of PS-OCT imaging was proposed, called an en-face birefringence map. From the birefringence map, the sensitivity, specificity, positive predictive value, and negative predictive values to detect dysplasia and early-stage cancer were 100.00%, 95.00%, 93.75%, and 100.00%, respectively, and the kappa value of these images between two investigators was 0.942. The mean size of malignant lesions detected in this study is 1.66 ± 0.93 mm. This pilot animal study validates the use of PS-OCT to detect small and early-stage oral malignancy with high accuracy and consistency.

## 1. Introduction

Oral cancer is the sixth most common cancer in the world, accounting for an estimated 300,000 new cases and 145,000 deaths in 2012, and 702,000 prevalent cases over five years, and the incidence has been increasing persistently [1]. Despite the advancement in cancer therapy, the survival rate of oral cancer has remained flat in the last 50 years because of delayed diagnosis [2]. Early diagnosis is difficult under the current screening program by visual examination, and up to 50% of cancers are not detected until the disease is quite advanced [3]. The biggest challenge of cancer screening is “field cancerization” in the oral cavity, which means exposure to carcinogens, like cigarette smoking or alcohol drinking, can produce multiple potentially malignant disorders (PMDs), like oral erythroplakia, leukoplakias, and lichenoid. Most PMDs are benign, but the mean overall malignant transformation rate is as high as 12.1% if dysplasia presents [4]. Although several commercialized adjunctive tools, such as toluidine blue, chemiluminescence, or tissue fluorescence imaging have been used to help identify PMDs, their use in diagnosing dysplastic lesions remains controversial because of high false-positive rate [5]. Until now, there has been a lack of useful tool to detect dysplastic PMDs with good accuracy.

Optical biopsy in vivo is a hot research topic to diagnose early cancers, and optical coherence tomography (OCT) is one of its potential tools. The mechanism of OCT is based on Michelson interferometry with low coherent light. It can give label-free, radiation-free, real-time, cross-sectional tomography images and three-dimensional (3D) reconstruction images with high resolution (10–20 μm) and adequate tissue penetration depth (1.5–2 mm). These properties are useful in the diagnosis of early cancer and the monitoring of tumor treatment. In oral cancer, studies have used OCT to diagnose early cancers in animals and humans. Wilder-Smith et al. had first used intensity OCT to image oral malignancies in golden Syrian hamster cheek pouches with 80% agreement between the OCT imaging and histology [6]. In following animal and human studies, structural variables by intensity OCT, including changes in keratin, epithelial, subepithelial layers, and the identification of the basement membrane (BM), were commonly used to differentiate benign or malignant lesions. For example, Tsai et al. used three OCT indicators, including the epithelium thickness, the standard deviation of an A-mode scan intensity profile in the epithelium layer, and the decay constant of the spatial-frequency spectrum of the A-mode scan profile, to differentiate benign and malignant oral lesions. They found that intensity OCT had a sensitivity of 82% and specificity of 90% to differentiate normal, mild dysplasia, and moderate dysplasia [7,8,9]. Hamdoon et al. had similar results, with a sensitivity of 85% and specificity of 78%. They found BM to be a critical landmark, but the interobserver agreement was only moderate. The commonly used marker of epithelium thickness was also variable in the oral cavity, and it has been difficult to use in the judgment of benign or dysplastic lesions [10].

Until now, the use of intensity OCT to differentiate oral lesions by structural changes alone has still been controversial, especially in dysplasia. These study results were not translated into clinical use. Neogengiogenesis is another important marker to differentiate benign and malignant lesions [11,12,13]. Recently, our group published the OCT angiography (OCTA) technique to observe neoangiogenesis in oral cancer development. By OCTA, the growth and changes of intraepithelial papillary capillaries loop (IPCL) during cancer development can be imaged and quantified in vivo. These results are complementary to conventional intensity OCT to improve the diagnostic accuracy, especially in stages of hyperplasia, dysplasia, and carcinoma in situ, in which only variable epithelial thickness can be observed by intensity OCT [14,15]. Nevertheless, the demand for stable and high-quality imaging in OCTA still limits its use in an oral cancer screening program if a large scanning area is needed.

Stromal change, an important factor in controlling malignant transformation, tumor differentiation, and invasiveness in oral cancer development, has captured researchers’ attention in recent years [16]. These changes, including the proliferation of tumor fibroblast cells, changes in collagen components, and other extracellular matrices, have been studied. The morphological change of stroma can be easily seen in conventional histology, like the change of core of papilla [17]. The changes in collagen fiber’s diameter, orientation, density, and packing during malignant transformation have also been studied in polarized light microscopy with Picrosirius red stain [18,19]. However, most of these studies are ex vivo and cannot be translated into clinical diagnosis. Recently, the evolution of second harmonic generation (SHG) imaging provides a chance to image stroma in vivo [20], but the development is still limited by small field-of-view (FOV) and expensive instruments. The studies linking oral cancer to SHG are also rare [21]. Polarization-sensitive OCT (PS-OCT), first demonstrated by Hee et al. [22], is an imaging modality that uses polarization interferometry with broadband light sources to obtain a micrometer scale, cross-sectional structure, and collagen fiber-enhanced images of biological tissues in vivo with a high scanning speed and large FOV. The interest in using PS-OCT to image the stoma of oral mucosa in vivo has increased recently [23,24,25]. Since collagen fibers, the main component of the stroma, are birefringent, changes in collagen fibers’ diameter, orientation, density, and packing during cancer development are expected to be detected by PS-OCT from changes of polarization parameters. This is a pilot animal study to use PS-OCT to detect oral dysplastic and early cancerous lesions. This study aims to propose a new analytic process of PS-OCT imaging into an en-face birefringence map to present dysplastic lesions intuitively, which can be easily used in a screening program to validate the accuracy of this new method.

## 2. Results

### 2.1. Animal Protocol and Histology Results

**Figure 1:**
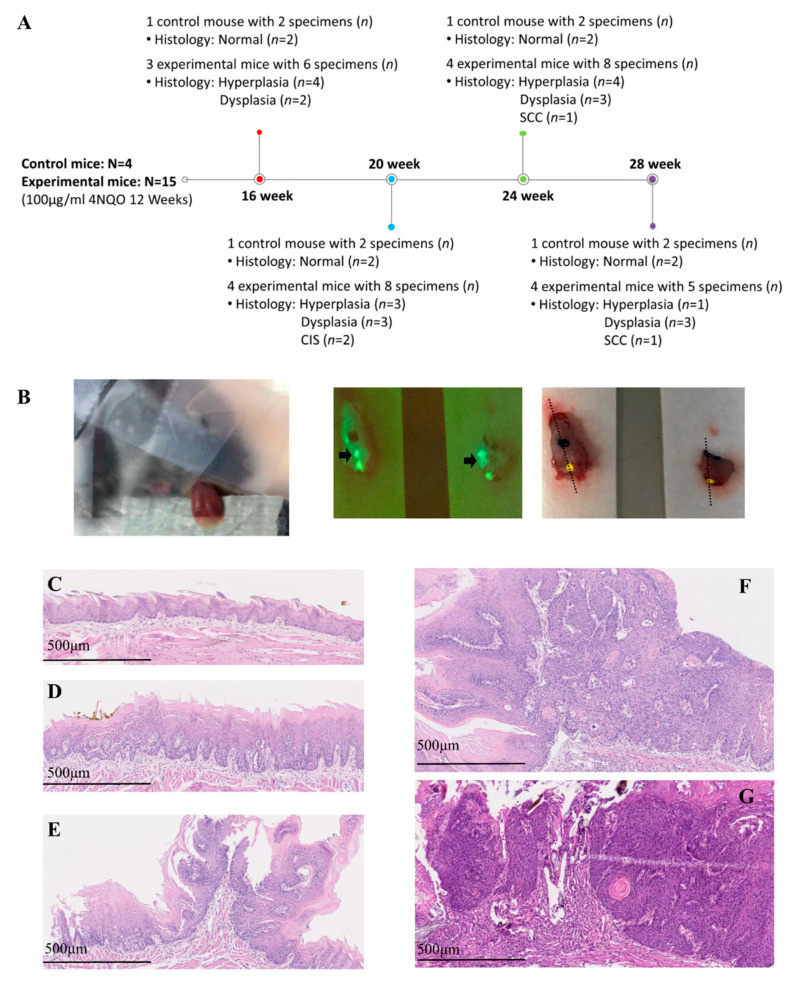
(**A**) Experimental schedule, number of mice (*N*), number of specimens (*n*), and histology results. (**B**) Polarization-sensitive optical coherence tomography (PS-OCT) scanning in vivo of the anterior tongue (left picture); fluorescence imaging to identify the green area, where indicates a potential tumor (middle picture, black arrow); and inking of the potential tumor area and histological sectioning on the black dotted line (right picture). (**C**–**G**) Histological definition of benign lesion: normal (**C**) and hyperplasia (**D**); histological definition of malignancy: dysplasia (**E**), carcinoma in situ (**F**), and squamous cell carcinoma (**G**).

For collecting sequential images from normal, hyperplasia, and dysplasia to cancer, the experiment was performed at different times: weeks 16, 20, 24, and 28 after the commencement of cancer induction. One control mouse and four experimental mice were enrolled in each experiment, and two specimens (anterior and posterior part of the tongue) were collected from each mouse. However, one experimental mouse on week 16 died during anesthesia. On week 28, a large tumor was found on the middle tongue of three mice; thus, the tongue cannot be divided into anterior and posterior parts in these three mice. Overall, four control mice and 15 experimental mice were enrolled in this study. Thirty-five tongue specimens (*n*) from these 19 mice (*N*) were collected and evaluated by PS-OCT and histology. The histological findings of these 35 specimens at different times are shown in Figure 1A. A total of 20 benign lesions and 15 malignant lesions (11 dysplasia, 2 carcinoma in situ (CIS), and 2 squamous cell carcinoma (SCC)) were found in histology.

### 2.2. PS-OCT Imaging and Birefringence Maps in Benign Lesions (Normal and Hyperplasia)

**Figure 2:**
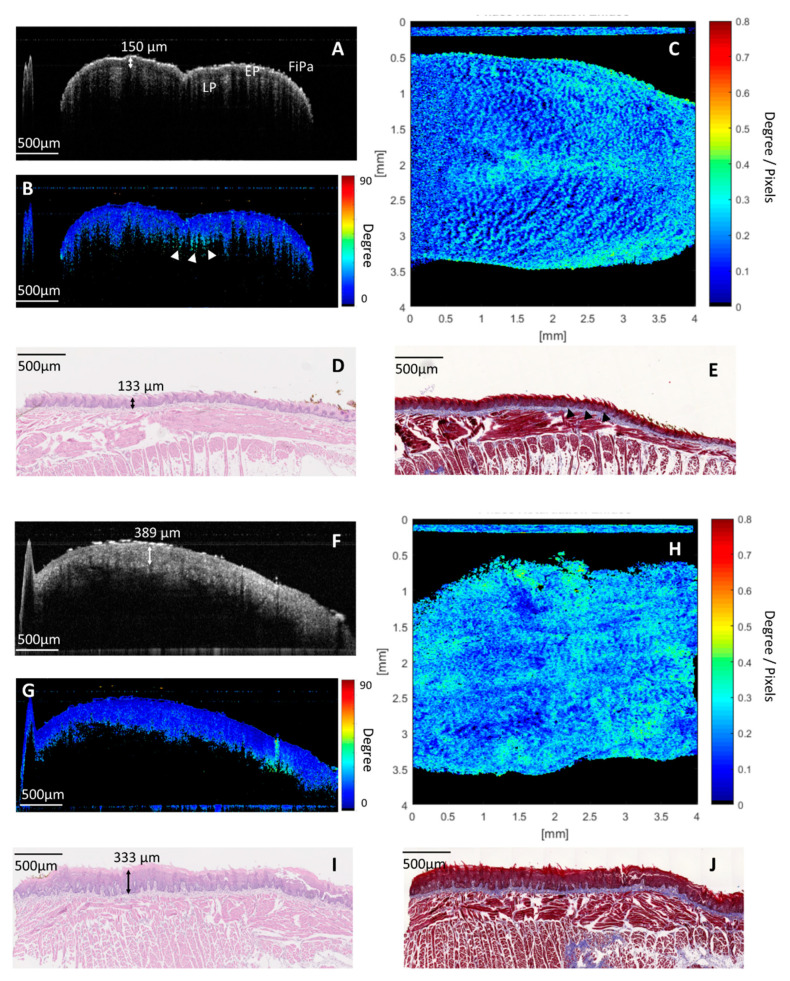
Images of the normal dorsal surface of the anterior tongue: (**A**) cross-sectional intensity optical coherence tomography (OCT) image. The microstructure of mucosa is shown, including filiform papilla (FiPa), the epithelium (EP), and the lamina propria (LP). (**B**) Cross-sectional cumulative PS-OCT image. The EP had low retardation. Some high retardant projection was seen beneath the EP (white arrowheads), where it is compatible with the collagen-abundant core of the papilla between the boundary of the EP and LP with Masson’s trichrome staining (black arrowheads in Figure 2E). (**C**) En-face birefringence map. Regularly arranged bright spots, where there are more birefringent agents like in the core of the papilla area, stand out against the homogeneous dark blue background. (**D**,**E**) Corresponding histology with hematoxylin and eosin (H&E) staining and Masson’s trichrome staining. Images of the dorsal surface of the anterior tongue with hyperplastic change: (**F**) cross-sectional intensity OCT image. An increased thickness of the EP was noted. (**G**) Cross-sectional cumulative PS-OCT. The thick EP showed low retardation. (**H**) En-face birefringence map. Blurred and irregular distribution of bright spots against a dark blue background. (**I**) Corresponding histology with H&E staining. Hyperkeratosis and increased thickness of the EP was noted. (**J**) Corresponding histology with Masson’s trichrome staining. The proliferation and irregular distribution of the collagen-abundant core of the papilla beneath the EP was seen.

Figure 2A–E shows an example of images in normal histology. In a cross-sectional intensity OCT image (Figure 2A), the microstructure of the dorsal surface of the anterior tongue, including filiform papilla, the epithelium (EP), and lamina propria (LP), can be seen clearly. The thickness of the EP was about 150 µm, which corresponds to the histology (EP: 133 µm in Figure 2D). Figure 2B showed a corresponding cross-sectional, cumulative PS-OCT image. Low retardation was seen in the EP. Some high-retardant projection was found beneath the EP (white arrowheads in Figure 2B), where it is compatible with collagen abundance in the core of the papilla at the boundary of the EP and LP in Masson’s trichrome stain (black arrowheads in Figure 2E). Figure 2C presented an en-face birefringence map of the dorsal surface of the anterior tongue. The bright spots indicate areas with more birefringent materials, such as those that exist in a collagen-abundant core of the papilla. In a normal birefringence map, the bright spot was regularly distributed in the dark blue background.

Figure 2F–J shows an example of images in hyperplastic change. From a cross-sectional OCT intensity image (Figure 2F), increased thickness of the EP up to 389 µm with a preserved LP was noted. In the corresponding cross-sectional cumulative PS-OCT image (Figure 2G), the thick EP layer showed low retardation. In the en-face birefringence map (Figure 2H), the distribution of bright spots became irregular. Some areas had crowded bright spots, and some areas had sparse bright spots. But no significant lesions with significant demarcation lines were found. The image also became more blurred than in normal histology. From histology with hematoxylin and eosin (H&E) staining (Figure 2I), hyperkeratosis in hyperplastic condition was noted, which may be the cause of the blurred image in the OCT. From histology with Masson’s trichrome stain, proliferation and irregular distribution of the core of the papilla beneath the EP were found (Figure 2J), which may have caused irregular distribution of bright spots in the birefringence map.

### 2.3. PS-OCT Image and Birefringence Map in Malignant Lesions (Dysplasia and Early-Stage Cancer)

**Figure 3:**
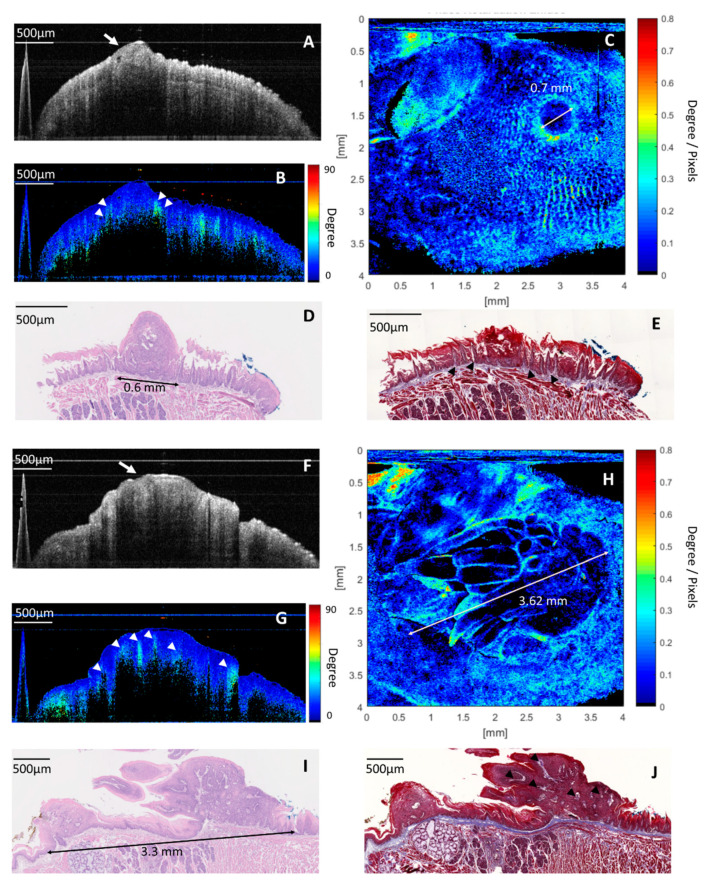
Images of the dysplastic lesion: (**A**) cross-sectional intensity OCT image. A hypo-intensity, a protruding lesion was seen in the EP (white arrow). (**B**) Cross-sectional cumulative PS-OCT image. The protruding lesion showed low retardation, and some highly retardant spikes at the boundary of the EP and LP surrounding the protruding lesion (white arrowheads). (**C**) En-face birefringence map presented a 0.7 mm lesion, with low birefringence in the center and high birefringence in the periphery, the size of the tumor (0.7 mm) is consistent with the histology in Figure 3D (0.6 mm). (**D**) Histology with H&E staining revealed a 0.6 mm papilloma with the dysplastic change, which is compatible with the low-retardance protruding lesion in a cross-sectional PS-OCT image. The expanded and less-retardant dysplastic cell mass explains the low birefringence in the center of the tumor in Figure 3C. (**E**) Histology with Masson’s trichrome staining showed that the collagen--abundant elongated core of the papilla (black arrowheads) at the boundary of the EP and LP surrounding the peripheral of papilloma, which explains the high birefringence at the periphery of the lesion in Figure 3C. Images of the carcinoma in situ (CIS): (**F**) cross-sectional intensity OCT image. A thick, hypo-intensity tumor at the EP was seen (white arrow). (**G**) Cross-sectional, cumulative PS-OCT image. The tumor showed low retardation, and some prominent high-retardant spikes projected from the LP into the low-retardance EP were found (white arrowheads). (**H**) An en-face birefringence map reveals a 3.62 mm lesion with low birefringence in the background and a highly birefringent septum and capsule. (**I**) Histology with H&E staining revealed a 3.3 mm tumor with a full thickness of dysplasia of EP. (**J**) Histology with Masson’s trichrome staining showed that collagen abundance elongated the core of the papilla (black arrowheads) projected from the LP into the EP and separated the dysplastic cell mass. These changes were compatible with the en-face birefringence map, in which the low birefringence background (dysplastic cell mass) was surrounded and separated by a high birefringence capsule and septum (the elongated core of papilla).

Figure 3A–E shows an example of images in the dysplastic lesion on the dorsal surface of the posterior tongue. In cross-sectional intensity OCT image, a small protruding lesion arising from the EP was seen (white arrow in Figure 3A). In a corresponding cross-sectional cumulative PS-OCT image, this protruding lesion had low retardation. Some highly retardant spikes at the boundary of the EP and LP surrounding the periphery of the protruding lesion were noted (white arrowheads in Figure 3B). In histology with H&E staining (Figure 3D), a papilloma with dysplastic cells protruding from the EP was noted. This location of papilloma correlated to the low retardant, protruding lesion in the cross-sectional PS-OCT image. In histology with Masson’s trichrome staining, several elongated cores of the papilla with abundant collagen at the boundary of EP and LP surrounded the peripheral of papilloma (black arrowheads in Figure 3E). The elongated core of papilloma corresponded to the highly retardant spikes in a cross-sectional cumulative PS-OCT image. Although the dysplastic lesion can be found on the cross-sectional OCT images, it is difficult to define the whole tumor picture clearly in either intensity or in the PS-OCT image. In contrast, a round lesion with central low birefringence and peripheral high birefringence was seen clearly in the en-face birefringence map (Figure 3C). This change can be explained from histology and cross-sectional cumulative PS-OCT imaging. The central, low birefringence area was related to the expanded dysplastic cell with few retardant changes in depth. The peripheral high birefringence was related to the elongated, collagen-abundant core of the papilla at the periphery of the dysplasia lesion. The diameter of the dysplastic lesion measured by the en-face birefringence map was 0.7 mm, which was close to the diameter in the histology (0.6 mm).

The second example was a case of CIS on the dorsal surface of the posterior tongue (Figure 3F–J). A similar thick and hypo-intense tumor at the EP was noted in the cross-sectional intensity OCT image (white arrow in Figure 3F). In a cross-sectional cumulative PS-OCT image, the tumor showed low retardation, and some prominent, highly retardant spikes projecting from underlying LP into the thick, low-retardance tumor were noted (white arrowheads in Figure 3G). In the en-face birefringence map (Figure 3H), a 3.62 mm oval tumor with a low birefringent background and a high birefringent septum and capsule was found. In histology with H&E staining (Figure 3I), a 3.3 mm tumor with the full thickness of dysplastic cells of the lining epithelium was noted, which was compatible with the thick, low-retardance tumor in the cross-sectional cumulative PS-OCT image. The expanded and low-retardance dysplastic cell mass explains the low birefringent background in the en-face birefringence map. In histology with Masson’s trichrome staining, the elongated, collagen-abundant core of the papilla surrounded the periphery of the tumor, and also projected from the LP into the center of the tumor (black arrowheads in Figure 3J). These projections separated the dysplastic cell mass and formed a highly birefringent septum and capsule in the en-face birefringence map.

### 2.4. Accuracy of En-Face Birefringence Map to Detect Dysplasia and Early Cancerous Lesion

**Figure 4:**
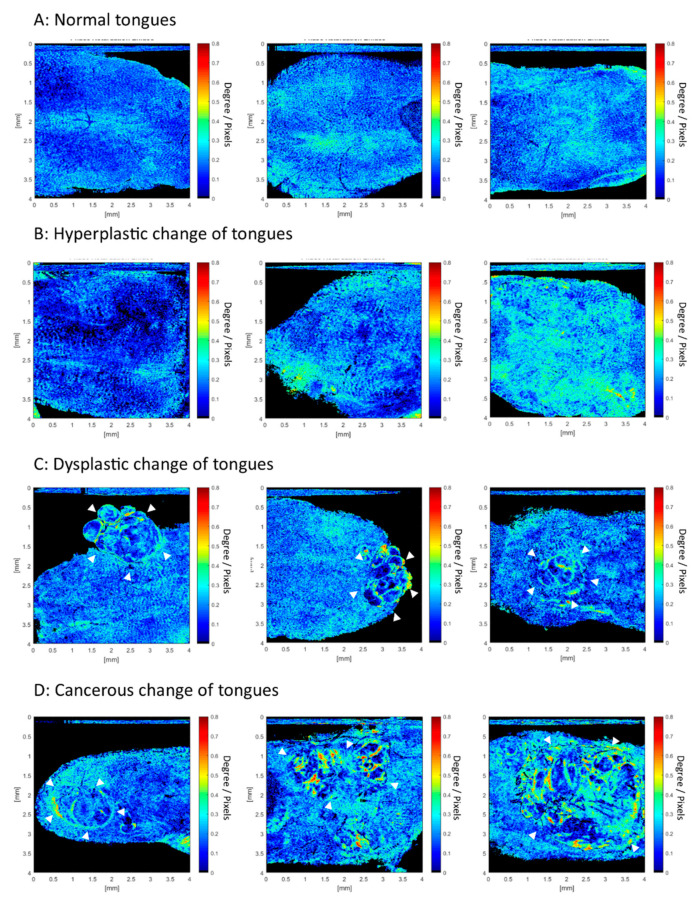
En-face birefringence maps from normal to cancers. (**A**) Bright spots against a dark blue background were seen in a normal tongue. (**B**) Crowded and blurred bright spots against a dark blue background in hyperplastic change. (**C**,**D**) Apparent malignant lesions (white arrowheads), with a low birefringence background and highly birefringent capsule and septum that can be seen clearly.

Learning from Figure 2 and Figure 3, two independent researchers read a total of 35 en-face birefringence maps separately. The results of these images were classified into benign and malignant (presence of dysplasia or early cancerous lesion). If a malignant lesion was suspected, the diameter of the lesion was measured from the birefringence map. Finally, only one image had a contradicting result between two researchers. The inter-observer kappa value was 0.942. After the discussion, this contradictive image was classified as malignant. Table 1 shows the results of histology and the en-face birefringence map of all specimens. The mean diameter of the tumor measured from the en-face birefringence map was 1.66 ± 0.93 mm, which was similar to the diameter measured in histology with H&E staining (1.47 ± 0.89 mm). The sensitivity, specificity, positive predictive value, and negative predictive value of using the en-face birefringence map to detect malignant lesions was 100%, 95%, 93.75%, and 100% (Table 2). Only one benign case in histology was incorrectly classified as malignant. Figure 4 presented more examples of en-face birefringence maps in our study. In a normal tongue (Figure 4A), the en-face birefringence map showed regularly arranged bright spots against a homogeneous dark blue background. In hyperplastic change (Figure 4B), irregular distribution of the bright spots with blurred images are seen, which are consistent with Figure 2H. In the dysplastic and cancerous images (Figure 4C,D), noticeable lesions with low birefringence in the central and high birefringent septum and capsule can be seen. It is easy and intuitive to identify these lesions from the en-face birefringence maps. The corresponding white light image of tongues in Figure 4 is shown in Figure S1.

**Figure 5:**
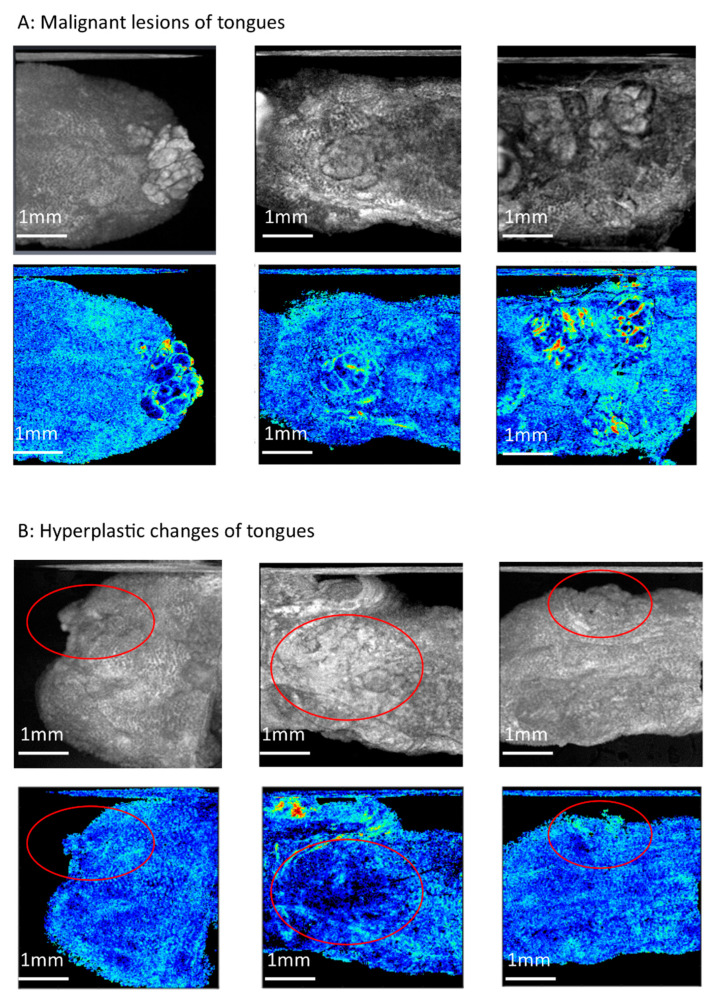
Compare en-face intensity images to the en-face birefringence maps. (**A**) Malignant lesions of tongues. The tumor can be seen clearly in these two en-face images (**B**) Hyperplastic changes of tongues. In en-face intensity images (upper pictures), the heterogeneous area was seen (circle area), which mimic the tumor. In corresponding en-face birefringence maps, the distribution of bright spots in the circle area without significant demarcation lines indicates hyperplastic change without a tumor.

To compare the efficacy of en-face birefringence maps to conventional intensity images, the averaged intensity projection in the same database was performed to present en-face intensity images. The sensitivity, specificity, positive predictive rate, and negative predictive rate of en-face intensity images to detect oral malignancy was 100%, 85%, 83.3%, 100%, respectively, and 100%, 75%, 75%, 100%, respectively, for the two investigators. The kappa value in these two investigators was 0.89. The higher false-positive predict rate was found in en-face intensity images. Figure 5 showed some examples. In malignant lesions (Figure 5A), a heterogeneous tumor can be seen clearly in the en-face intensity image because of hyper-scattering on the tumor surface. In hyperplastic tongues, hyper-scattering sometimes happened because of hyperkeratosis and uneven epithelial thickness (Figure 5B). This would make a heterogeneous area in the en-face intensity image that mimics the tumor (circle area in Figure 5B). However, in the corresponding en-face birefringence maps, the distribution of the bright spots in the circle area without significant demarcation line to the surrounding mucosa was seen, which is very different from the tumor changes in Figure 5A and indicated the hyperplastic changes.

## 3. Discussion

This is the first study to use PS-OCT to image different stages of oral pathogenesis from normal to cancer in vivo. From benign to malignant lesions, the increased thickness of the low-retardance epithelial layer is shown in Figure 2 and Figure 3. These results are compatible with a previous study that determined that dysplastic epithelial cells had low retardation [26]. During malignant transformation, elongated projection of the core of the papilla from the LP into the tumor has been noted in histology, which presents highly retardant spikes seen in cross-sectional, cumulative PS-OCT (Figure 3B,G). These retardant changes in PS-OCT are consistent with previous studies using Picrosirius red staining and polarized light microscopy, which have shown densely packed collagen fibers with high birefringence surrounded the tumor islands in well-differentiated tumors [18,19,27,28].

In contrast to the conventional, histology-based method, our study not only provides an in vivo image to see the stromal change, but also proposes a new insight into the en-face birefringence map for detecting diminutive malignant lesions. Comparing to the traditional cross-sectional view in histology, the en-face view is parallel to the organization of collagen fiber, and it is easier to present changes in those fibers. The linear curve fitting method used in this study also increases the contrast of birefringent changes. For example, in Figure 4A, the normal core of papilla distribution can be seen clearly in the en-face birefringence map. This is due to more birefringent collagen and vessels in the core of the papilla area. After the linear curve fitting, the minimal difference between a low-birefringence EP and the highly birefringent core of the papilla becomes apparent. Through en-face presentation of the fitting results, we can easily find out the area that has abnormal birefringent changes, like those in Figure 4C,D. Expanded dysplastic cells in the EP and the elongated core of the papilla are two histological features of malignant transformation. These changes result in significant differences between the low-retardance dysplastic cells and the highly retardant core of the papilla, and are presented well in en-face birefringence maps. The tumor diameter can also be measured easily in the en-face birefringence map. The measurement bias between the en-face birefringence map and histology is only 185 ± 121 µm in our study. Although we cannot claim that the en-face birefringence map can predict tumor margin accurately in this study, this result still inspires the potential use of PS-OCT to define tumor margins in the future, as has been done in studies of breast cancer [29,30].

Presence of dysplasia is the most critical predictor of oral cancer [31]. However, there has been a lack of tools to detect dysplasia accurately in vivo until now. Up to 50% of oral cancers cannot be detected by visual examination until the disease is quite advanced [32]. Many efforts had been made to use intensity-based OCT to detect oral dysplastic lesions, but the results are controversial. The variation of normal epithelial thickness in different locations of the oral cavity and the fluctuant epithelial thickness in dysplasia also makes it difficult to differentiate the benign and dysplasia lesion by intensity OCT [9,33]. From our previous studies and experience [14,15], we have also found it challenging to differentiate hyperplasia, dysplasia, and carcinoma in situ by intensity OCT alone, because only variable epithelial thickness can be observed. Recently, some evidence has shown that stromal changes might be present early in dysplasia or early-stage cancer [34,35,36]. The use of PS-OCT to detect the stromal change in the diagnosis of dysplasia or early cancers had initial promising results in some studies [26,37], but the evidence is still weak. In our study, we found that the sensitivity, specificity, positive predictive value, and negative predictive value of the en-face birefringence map to detect dysplasia and early-stage cancer was 100%, 95%, 93.75%, and 100% (Table 2). Compared to the conventional, intensity-based method, the en-face birefringence maps also reduce the false-positive prediction rate because of less interference by hyper-scattering. In this study, 13 of the 15 malignant lesions were dysplasia and CIS. The mean size of the malignant lesion was 1.47 ± 0.89 mm, and the smallest dysplastic lesion was only 0.46 mm. Although no cut-off value of retardation changes was available to diagnose in this study, it is still straightforward to distinguish the malignant lesions from the surrounding normal mucosa in the birefringence map by the naked eye because of the enhanced stromal contrast. The inter-observer agreement of en-face birefringence maps between two researchers is high (kappa value = 0.942). The high sensitivity and consistency in our study support the potential use of PS-OCT. This technique can be complementary to intensity OCT to increase diagnostic accuracy, especially in a small dysplastic lesion, which has subtle structural changes. After combing the OCT angiography technique in our previous work [14,15], the detection, differentiation, and staging of oral dysplasia and early-stage cancer can be achieved by one OCT scan.

There are several limitations to our study. All of the dysplastic or cancerous lesions in this study were well-differentiated protruding tumors because of the limitations of the 4-Nitroquinoline-1-oxide (4-NQO) animal model. In a previous ex vivo study using polarized light microscopy with Picrosirius red staining, it was found that different differentiations of oral cancer had different changes in collagen fibers [18]. Thus, it is expected that the birefringent pattern in poorly differentiated cancer would be different from well-differentiated cancer in this study. Lack of flat lesions is another weak point of this animal model. Theoretically, the retardation is less effected by epithelial thickness because of very low birefringence in epithelial dysplastic cells. However, this still needs cases to validate the usability of PS-OCT in the flat or depressive lesion. The pathogenesis of oral cancer in humans is also more complicated than the animal model, like submucosal fibrosis and human papillomavirus (HPV) infection. Besides, this study used a single-mode, fiber-based PS-OCT system, which is simple and compact, that helps with the robustness of the system. However, compared with the conventional intensity OCT system, the requirements of the polarization diversity detection module and dual-channel data acquisition still increase the total cost. In conclusion, this is a pilot study to propose the potential use of PS-OCT in the diagnosis of oral dysplasia and early-stage cancer. Further studies to collect more PS-OCT images in different human oral pathogenesis should be conducted to validate its clinical use.

## 4. Materials and Methods

### 4.1. Swept-Source, Polarization-Sensitive Optical Coherence Tomography (SS PS-OCT) Imaging System

**Figure 6:**
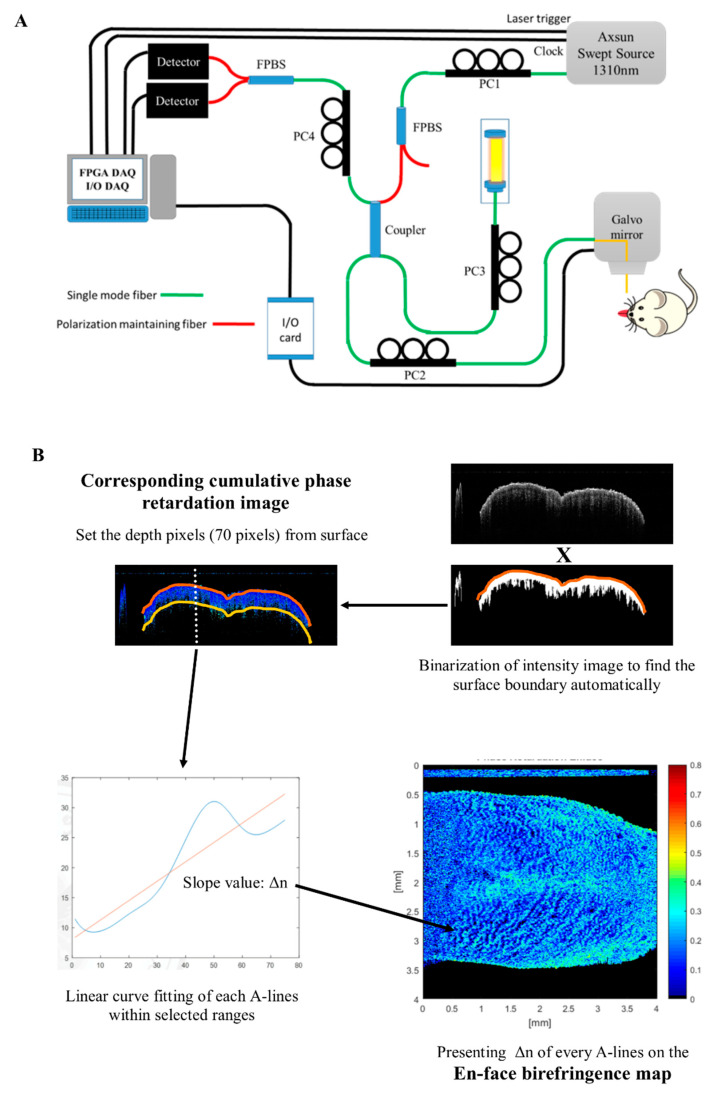
(**A**) Setup of the swept-source, polarization-sensitive optical coherence tomography (SS PS-OCT) imaging system (PC: polarization controller; FPBS: fiber polarization beam splitter). (**B**) Process of the en-face birefringence map: a gray-level thresholding algorithm was used to binarize the intensity image and find out the upper border of the surface automatically. After determining the surface, the fixed depth (70 pixels, about 410 µm) was set to the corresponding cumulative phase retardation image. Then, linear curve fitting was performed between the surface and selected depth to each A-lines. The slope value (Δ*n*) of each A-line was presented in an en-face birefringence map. (4 × 4 mm in the *x*–*y* plane).

Figure 6A shows the platform setup of the swept-source (SS) PS-OCT system. The light source was a swept-source laser (AXSUN Technologies Inc., Billerica, MA, USA) with a central wavelength of 1310 nm, spectral bandwidth of 100 nm, and sweeping speed of 100 kHz. The swept-source contains a built-in Mach-Zehnder Interferometer that provides a frequency clock for the laser to resample each record from the sample signal to an equidistant spacing in the frequency. A fiber polarization beam-splitter (FPBS) with a light polarization controller (PC), PC1, was used to get pure, linearly polarized light and maximize its output power. The SS PS-OCT system was constructed with all single mole fibers, as proposed by our lab in 2014 and 2018 [38,39]. A 50/50 coupler was arranged to split the light power into 50% incident power for the reference mirror (with an attenuator), with the remaining 50% incident power (~7 mW) focused on a scanning spot. PC2 was adjusted to generate a circularly polarized light in the sample arm, and the combination of PC3 and PC4 was used to balance the power of the reflected reference light until it arrived equally at the two detectors. After the recombination of the reflected beams from the sample and reference arms, the horizontally and vertically polarized signals were further split by an FPBS and detected by two photodetectors (PDB470C, Thorlabs Inc., Newton, NJ, USA) to calculate polarization changes. Finally, the fringe signals (including the horizontal and vertical polarization channels) from the OCT interferometer and the output of the clock signal were simultaneously recorded by a high-speed analog-to-digital (A/D) converter operating at 100 M samples/s with 14-bit resolution (AlazarTech, Pointe-Claire, QC, Canada, Model ATS9440). The maximum image acquisition rate was 100 frames per second (1000 × 1024 pixels in the *x*–*z* plane). The axial resolution of the imaging system in the air was measured as ~15 µm by using a mirror with an attenuator as a sample. A 1951 USAF resolution test target (Edmund Optics, Barrington, New Jersey, USA) was used as the standard resolution test sample for measurement of the lateral resolution, confirming that the lateral resolution of the OCT setup was approximately 7 µm.

### 4.2. Process of PS-OCT Imaging and Build an En-Face Birefringence Map

Data were post-processed to get intensity and retardation images using MATLAB (MathWorks, Natick, MA, USA). Images were shown in two-dimensional cross-sectional (*x*–*z*) planes and an en-face (*x*–*y*) plane. After the acquisition of the interference signal, the shape of the spectrum was modified with dispersion compensation to achieve better resolution [40]. The A-scan signal obtained by inverse Fourier transform of the acquired fringe data can be expressed as

(1) $$FT^{-1}\{S_{x}(k)\}\to S_{x}(z)=\sqrt{R_{s}(z)}\sin(\delta (z))\exp(i\phi_{x})\;\mathrm{and}$$

(2) $$FT^{-1}\{S_{y}(k)\}\to S_{y}(z)=\sqrt{R_{s}(z)}\cos(\delta (z))\exp(i\phi_{y})$$

where *x* and *y* denote the horizontal and the vertical polarization channel, respectively; $R_{s}(z)$ is the sample reflectivity, while both the sin(δ(z)) and cos(δ(z)) terms are the retardation moduli modulated by the birefringence of the measured samples. Then the two-dimensional cross-sectional structural images ($R_{s}(z)$) and retardation images (δ(z)) were calculated by using the amplitude of the A-scan signal, as prescribed for traditional bulk optics-type, circular state PS-OCT [41]. The intensity images were displayed on a logarithmic grayscale in a two-dimensional (2D) format. The cumulated phase retardation was presented in a 2D color image (range from 0° to 90°). A 3D reconstructed data set included a volume of 4 mm × 4 mm × 6 mm, corresponding to 1000 × 1000 × 1024 pixels in the *x*, *y*, and *z* directions.

A schematic of the analysis process of the en-face birefringence map is shown in Figure 6B. First, a gray-level threshold algorithm was used to binarize the cross-sectional intensity structural image, and to find out the upper border of the surface automatically. After determining the surface, the fixed depth (70 pixels, about 410 µm) was set to include epithelial and lamina propria layers. These positions were recorded to correspond to the cross-sectional phase retardation image. Birefringence coefficient (Δn) of each A-line could then be calculated by linear least-squares fitting through the averaged δ(z) data of four nearby A-lines, and then its slope could be determined from the following formula: δ = (360/2π)·*k*_0_·*d*·Δ*n*, where *k*_0_ is the wave vector and *d* is the thickness of the fitting range (i.e., 70 pixels). The *R*-squared of each fitted retardation A-line was also calculated within the selected range to show the correlation between measured retardation change and its linear fitting results. The slope value with *R*-squared > 0.7 was presented in a colored en-face map; other slope values with *R*-squared < 0.7 were set to be zero and colored in black.

### 4.3. Animal and Experimental Protocol

K14-EGFP-miR-211-GFP transgenic mice, generated by Chang et al., were used in this study. The advantage of this model is a higher potential for oral cancer induction. The diminutive dysplastic lesions can also be identified easily on fluorescent imaging by increased expression of K14-EGFP-miR-211-GFP [42].

4-Nitroquinoline-1-oxide (4NQO), a water-soluble quinoline derivative that forms DNA adducts and induces intracellular oxidative stress, can result in mutations and breakage of the DNA strand. It can be a carcinogen to induce oral squamous cell carcinoma (SCC), similar to the carcinogenesis provoked by tobacco exposure. The pathological pathway also resembles human oral SCC, including the progression from hyperplasia, dysplasia, and carcinoma in situ (CIS) to SCC, and can sequentially develop as time goes on after induction [43].

To induce tumorigenesis, 100 µg/mL of the water-soluble carcinogen 4NQO (Sigma-Aldrich, St. Louis, MO, United States) was added to the drinking water of six-week-old, K14-EGFP-miR-211-GFP transgenic mice for 12 weeks. The experiments were performed in weeks 16, 20, 24, and 28 after the commencement of cancer induction (Figure 1A). The steps of the experiment were as follows: (1) the mouse was anesthetized by intraperitoneal injection of 2,2,2 tribromoethanol (200 mg/kg); (2) the anterior part of the tongue was gently extended, fixed, and scanned by PS-OCT in vivo (Figure 1B, left picture); (3) after image acquisition, the mouse was sacrificed, the whole tongue was extracted and divided into an anterior and posterior part, and the posterior part of the tongue was scanned immediately by PS-OCT ex vivo, using the same protocol with the anterior tongue; (4) two parts of the severed tongue were scanned by a fluorescent system (LT-9900 Illumatool bright light) to identify the green area, which indicated a potential tumor area (the expression of K14-EGFP-miR-211-GFP) (black arrow on Figure 1B, middle picture); (5) the green area was inked, and histological sections were made at the inked area (black dotted line on Figure 1B, right picture), after which the histological slides were stained with H&E and Masson’s trichrome staining; (6) the slides were assessed by two experienced pathologists who were blinded to the PS-OCT image, using the definition of histological changes based on the binary oral epithelial dysplasia grading system [44]. To simplify the reading, we integrated low-grade dysplasia into hyperplasia and named high-grade dysplasia as dysplasia in this study. The histological results were further classified into benign (normal, hyperplasia) (Figure 1C,D) or malignant (dysplasia, CIS, and SCC) (Figure 1E–G) to compare to the PS-OCT images. (7) The en-face birefringence maps were read by two researchers independently, who were blinded to the histological results. The images were also classified into benign or malignant. Finally, (8) the results of en-face birefringence maps were compared to histology. The sensitivity, specificity, positive predictive value, and negative predictive value of the en-face birefringence map to detect malignant lesions were calculated.

All experiments were approved by the Institution for Animal Care and Use Committee at National Yang-Ming University (ethical code: #1070216).

## 5. Conclusions

PS-OCT is noninvasive and real-time imaging to detect minimal birefringent changes in oral mucosa in vivo. The new process of the en-face birefringence map has high sensitivity and accuracy in detecting diminutive dysplastic and early-stage cancerous oral lesions. The potential use of this technique to identify tumor margins is also expected. Further study to combine intensity OCT and PS-OCT will be needed in humans to validate its clinical use.

## Figures and Tables

**Table 1:** Results of histology and en-face birefringence map in all specimens.

Mice	Histology	Maximal Tumor Diameter by Histology	Maximal Tumor Diameter by Birefringence Map
16 weeks			
Control			
Ant. Tongue	Normal	Nil	Nil
Pos. Tongue	Normal	Nil	Nil
E-Mouse 1			
Ant. Tongue	Hyperplasia	Nil	Nil
Pos. Tongue	Hyperplasia	Nil	Nil
E-Mouse 2			
Ant. Tongue	Dysplasia	1.41 mm	1.58 mm
Pos. Tongue	Dysplasia	0.60 mm	0.70 mm
E-Mouse 3			
Ant. Tongue	Hyperplasia	Nil	Nil
Pos. Tongue	Hyperplasia	Nil	Nil
20 weeks			
Control			
Ant. Tongue	Normal	Nil	Nil
Pos. Tongue	Normal	Nil	Nil
E-Mouse 1			
Ant. Tongue	Dysplasia	1.26 mm	1.53 mm
Pos. Tongue	Dysplasia	1.26 mm	1.30 mm
E-Mouse 2			
Ant. Tongue	Hyperplasia	Nil	Nil
Pos. Tongue	Dysplasia	0.60 mm	0.70 mm
E-Mouse 3			
Ant. Tongue	Hyperplasia	Nil	Nil
Pos. Tongue	CIS	3.30 mm	3.62 mm
E-Mouse 4			
Ant. Tongue	Hyperplasia	Nil	Nil
Pos. Tongue	CIS	1.17 mm	1.51 mm
24 weeks			
Control			
Ant. Tongue	Normal	Nil	Nil
Pos. Tongue	Normal	Nil	Nil
E-Mouse 1			
Ant. Tongue	Hyperplasia	Nil	Nil
Pos. Tongue	Hyperplasia	Nil	0.70 mm
E-Mouse 2			
Ant. Tongue	Multifocal dysplasia	1.15 mm and 0.46 mm	1.31 mm and 0.60 mm
Pos. Tongue	Hyperplasia	Nil	Nil
E-Mouse 3			
Ant. Tongue	SCC	1.73 mm	1.81 mm
Pos. Tongue	Dysplasia	0.70 mm	0.78 mm
E-Mouse 4			
Ant. Tongue	Dysplasia	0.71 mm	1.07 mm
Pos. Tongue	Hyperplasia	Nil	Nil
28 weeks			
Control			
Ant. Tongue	Normal	Nil	Nil
Pos. Tongue	Normal	Nil	Nil
E-Mouse 1			
Ant. Tongue	Hyperplasia	Nil	Nil
Pos. Tongue	Dysplasia	2.20 mm	2.64 mm
E-Mouse 2			
Mid. Tongue	Dysplasia	2.44 mm	2.61 mm
E-Mouse 3			
Mid. Tongue	Dysplasia	1.30 mm	1.43 mm
E-Mouse 4			
Mid. Tongue	Squamous cell carcinoma (SCC)	3.23 mm	3.30 mm

Abbreviations: Ant, anterior; Pos. posterior; Mid, middle; E, experimental; Nil, no tumor.

**Table 2:** Accuracy of en-face birefringence map to detect dysplasia or early cancerous lesion.

Histology	Malignancy	Benign	Sensitivity	Specificity	PPV	NPV
	(Dysplasia–SCC)	(Normal–Hyperplasia)				
	(*n* = 15)	(*n* = 20)				
**Birefringence Map**						
**Malignancy**	**15**	**1**	**100%**	95%	93.75%	100%
Benign	0	19				

Abbreviations: PPV, positive predictive value; NPV, negative predictive value.

## Supplementary Materials

The following are available online at https://www.mdpi.com/2072-6694/12/9/2376/s1, Figure S1: Corresponding white light image of tongues in Figure 4.
