# Supplementary material for: Detection of Oral Dysplastic and Early Cancerous Lesions by Polarization-Sensitive Optical Coherence Tomography
Source: Cancers (Basel). 2020 Aug 22;12(9):2376. doi: 10.3390/cancers12092376 (PMC7564531; doi:10.3390/cancers12092376)
Supplement: Supplementary file 1 [file cancers-12-02376-s001.pdf]

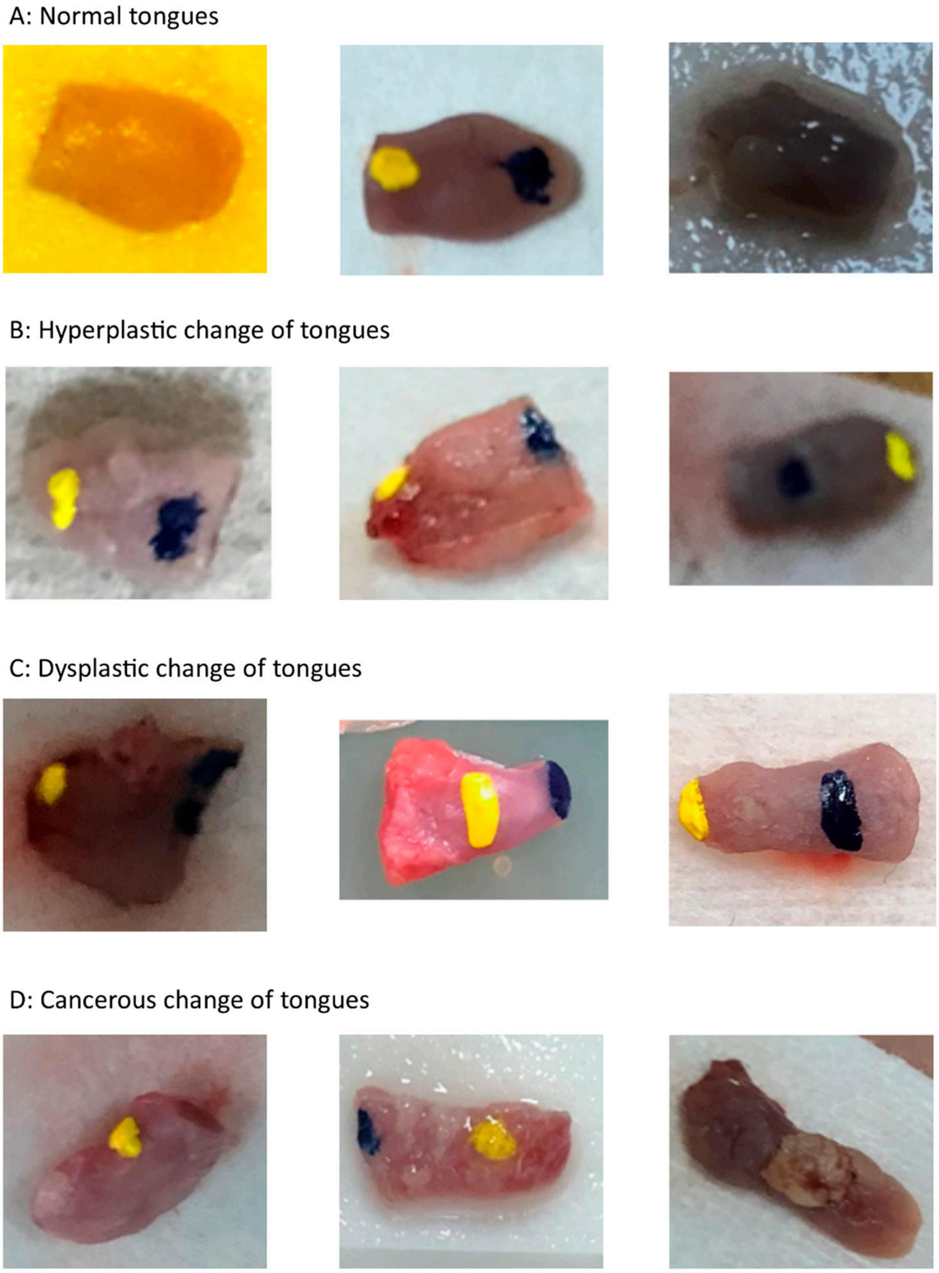

Figure S1. Corresponding white light image of tongues in Figure 4.
